# Musk gland seasonal development and musk secretion are regulated by the testis in muskrat (*Ondatra zibethicus*)

**DOI:** 10.1186/s40659-017-0116-9

**Published:** 2017-03-04

**Authors:** Tianxiang Zhang, Dong Peng, Lei Qi, Weixuan Li, Mengyuan Fan, Jiachen Shen, Liangliang Yang, Yihua Wang, Wenxia Wang, Xiaolong Hu, Ruibo Cai, Ran Zhou, Yuting Wei, Juntong Zhou, Shuang Yang, Defu Hu, Shuqiang Liu

**Affiliations:** 1 College of Nature Conservation, Beijing Forestry University, Beijing, 100083 People’s Republic of China; 2 College of Biological Science and Technology, Beijing Forestry University, Beijing, 100083 People’s Republic of China; 3 Zhonshan School of Medicine, Sun Yat-sen University, Guangzhou, 510080 People’s Republic of China

**Keywords:** Muskrat, Musk gland, Testis, AR, Seasonal development

## Abstract

**Background:**

The muskrat is a seasonal breeder. Males secrete musk to attract females during the breeding season. The testosterone binding to the androgen receptor (AR) in musk glands of muskrat may play an important role conducting the musk secretion process.

**Methods:**

The musk gland, testis and blood samples of musk rats are collected in both breeding and non-breeding seasons. Some part of the samples are kept in liquid nitrogen for transcriptome analysis and Western blotting test. Some part of the samples are kept in 70% alcohol for histology experiment, blood samples are kept at −20 °C for the serum testosterone measurement experiment.

**Results:**

This study demonstrates that the quantity of secreted musk, the volume of the musk glands, the diameter of the gland cells and AR expression are all higher during the breeding season than at other times (p < 0.01). StAR, P450scc and 3β-HSD expression in the Leydig cells of the testis were also higher during this season, as was serum testosterone. AR was also observed in the gland cells of two other musk-secreting animals, the musk deer and small Indian civet, in their musk glands. These results suggest that the testes and musk glands co-develop seasonally.

**Conclusion:**

The musk glands’ seasonal development and musk secretion are regulated by the testes, and testosterone plays an important role in the seasonal development of musk glands.

**Electronic supplementary material:**

The online version of this article (doi:10.1186/s40659-017-0116-9) contains supplementary material, which is available to authorized users.

## Background

The muskrat (*Ondatra zibethicus*) is a medium-sized, semi-aquatic rodent animal. The muskrat is a seasonal breeder, and males secrete musk from their musk glands to attract females during the breeding season. The breeding season begins in March and ends in October, lasting 8 months. Like those produced by musk deer (*Moschus berezovskii*) and the small Indian civet (*Viverricula indica*), the musk that male muskrats’ musk glands secrete during the breeding season is not only an important pheromone for attracting females [1].

The exploration of musk secretion mechanism is important to understand the seasonal change of musk secretion. Our previous study show the testis has seasonal change in muskrat, but the relationship of musk gland and testis is not clear. Prior research has suggested that musk gland development and function might be regulated by androgens [2, 3] produced by the testis under the control of the hypothalamus-pituitary-testis system [4, 5].

Androgens mediate a wide range of developmental and physiological responses [6, 7]. Testosterone as one of the most important functional androgen hormone, which is mainly produced by Leydig cells in testis and binds to the androgen receptor (AR), modulating gene transcription in various cells [8]. AR is expressed not only in male and female reproductive organs, but also in non-genital tissues [6, 9, 10].

Testosterone production in the Leydig cells is primarily mediated by the steroidogenic acute regulatory protein (StAR) [11, 12]. Cholesterol is converted to pregnenolone by the cytochrome P450 cholesterol side-chain cleavage (P450scc) enzyme [13]. Pregnenolone is next metabolized to androgens by 3β-hydroxysteroid dehydrogenase (3β-HSD) [14]. In the developed testis, Leydig cells (LCs) maintain high levels of P450scc and 3β-HSD and, in response to luteinizing hormone (LH), rapidly synthesize StAR and testosterone [15, 16]. Prior results showed that AR is expressed in musk glands, suggesting that the testis might regulate musk gland development. Here we studied the relationship between the musk glands and the testes in different seasons, to further understand the regulation of the development and function of the former by the latter.

## Methods

### Animals

Three adult male muskrats were obtained in the breeding season (April) and three in the non-breeding season (November) from Xinji Jinmu Muskrat Breeding Farm, Hebei, China. The six individuals were similar in size and weight. The musk glands were collected from one adult male musk deer dead of an accident (in July) at Fengxian Musk Deer Breeding Farm, Shanxi, China. The musk gland samples of the small Indian civet were collected from one adult male individual dead of an accident (in June) at Hengshan Wild Animal Breeding Farm, Anhui, China. All animals were treated in accordance with the National Animal Welfare Legislation. All experimental procedures were carried out in accordance with the guidelines established by the Beijing Forestry University. After fixation, the musk glands and testes were kept in 70% alcohol until used for immunohistochemistry. The lengths of the muskrats’ musk glands were measured and recorded. The contralateral musk gland of each muskrat was divided into small pieces and some of them were kept in liquid nitrogen for RNA-seq analysis and Western blotting test. The rest of the pieces were kept in ribonuclease inhibitor at 4 °C. Blood was centrifuged at 3000*g* for 20 min to separate serum from blood cells, which were collected and stored at −20 °C until used for hormone analysis.

### Musk secretion weight measurement

Ten adult male muskrats were selected for musk secretion measurement. The total musk weight in the breeding season of the selected muskrats was recorded, beginning on March 1. The measurement was made 3 times per half month. Measurements during the non-breeding season were taken in the same way, beginning on October 15.

### Histology

The musk gland and testis samples were dehydrated in an ethanol series and embedded in paraffin wax. Serial sections (4–6 μm) were mounted on slides coated with APES (3-aminopropyl-triethoxysilane). Some sections were stained with haematite hematoxylin (Solarbio) for observations of general histology.

### Immunohistochemistry

Serial sections of musk gland were incubated with primary polyclonal antibody (200 μg/ml, 1:200 dilution) against AR (Abcam) for 12 h at 4 °C. Serial sections of testis were incubated with primary polyclonal antibody (200 μg/ml, 1:200 dilution) against StAR (Santa Cruz Biotechnology), P450scc (Abcam) or 3β-HSD (Abcam) for 12 h at 4 °C. The sections were then incubated with a second antibody, goat anti-rabbit IgG conjugated to biotin and to peroxidase with avidin, using a rabbit ExtrAvidin staining kit (ZSGB-BIO), followed by visualizing with 0.5 mg 3,3-diaminobenzidine (Solarbio) solution in 1 ml of 0.05 M Tris–HCl buffer, pH 7.6, plus 0.4 μl H_2_O_2_.

### Western blotting

The musk gland tissues were kept at −80 °C. The samples were from three individuals in April and another three in November. Take approximately 0.1 g tissue from each individuals. Homogenize the tissue in a homogenizer containing 300 μl of 10 mg/ml PMSF stock and incubated on ice for 30 min while maintaining the temperature at 4 °C throughout all the procedures. Take 20 μl protein sample mixed with 5 μl loading buffer (final concentration: 32 mM Tris–HCl, pH 6.8, 12.5% glycerol (v/v), 1% SDS, and 31 μM β-mercaptoethanol) and denature it at 100 °C for 5 min. Separate the samples and marker (Fermentas, 10–170 kDa) on 12% polyacrylamide gels, and transferred onto PVDF membranes. The membranes were blocked in 5% non-fat dry milk and incubated with primary antibodies (rabbit anti-rat AR, 200 μg/ml, 1:2000 dilution) at room temperature for 60 min, washed in 0.1% Tween-20 containing buffer. Secondary incubation of the membrane was then carried out using a 1 mg/ml, 1:40000 dilution of goat anti-rabbit IgG tagged with alkaline phosphatase for 60 min.

### Hormone measurements

Serum testosterone was assayed by use of a testosterone ELISA kit (BNIBT). The operation was conducted according to the specification.

### RNA isolation and reverse transcription

The musk gland tissues were kept in RNA Fixer (Biomarker technologies, China) at 4 °C. The samples were from three individuals in April and another three in November. Total RNA was isolated using Trizol reagent (Qiagen, USA) according to manufacturer recommendations. RNA of checked quality was reverse transcribed into complementary DNA (Omniscript RT Kit, Qiagen, USA) following the manufacturer’s protocol.

### RT-PCR

The PCR conditions were 94 °C for 3 min, followed by 33 cycles at 94 °C for 30 s, 55 °C for 20 s, and 72 °C for 20 s using a melting curve program (increasing the temperature from 55 to 95 °C at 0.5 °C per 10 s) and continuous fluorescence measurement. The PCR primers used in this experiment were 5′-gagacagagtggacgggat-3′ and 5′-ggaggttacaccaaaggg-3′. Transcription of GAPDH gene was used as a reference. PCR products were electrophoresed on 1.0% agarose gels.

### RNA-seq analysis

Total RNA was isolated from musk gland tissues using Trizol reagent (Qiagen, USA), the quality of RNAs was determined by gel electrophoresis and spectrophotometry. Approximately 20 μg of total RNAs from two individuals in each season (April and November) was used for Illumina sequencing at Biomarker technologies (Beijing, China). All procedures for cDNA library construction were performed via the standard Illumina sample preparation protocol. Sequencing of the purified libraries were carried out on an Illumina GA-II (Illumina Inc., USA).

### Statistical analysis

In this study, the statistical comparisons were made with the Student *t* test and One-way analysis of variance.

## Results

### Musk secretion weight comparison

**Fig. 1:**
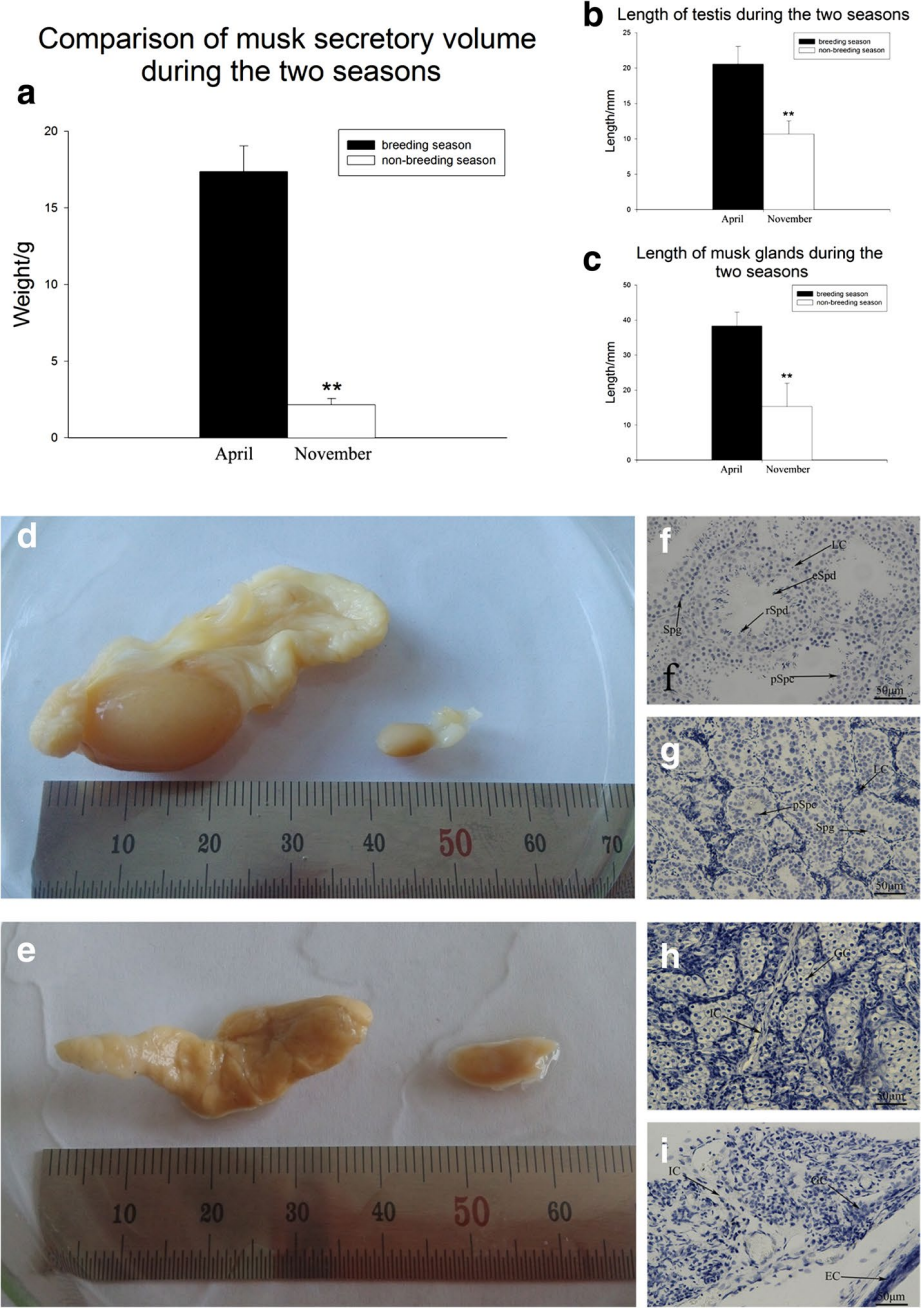
Morphological difference of musk glands and testis between the two seasons. **a** Comparison of muskrat musk secretion weight in the breeding and non-breeding seasons. The *left bar* represents the total musk secretion weight of 10 muskrats in March and April (17.36 ± 1.67 g). The *right bar* represents the total musk secretion weight of 10 muskrats in October and November (2.16 ± 0.40 g). The musk secretion weight in the breeding season was obviously greater than that in the non-breeding season (p < 0.01). *Different symbols* denote statistically significant values (**p < 0.01). **b** Comparison of testis lengths in the breeding and non-breeding seasons. The *left bar* represents the average testis length in April (20.6 ± 2.5 mm). The *right bar* represents the average testis length in November (10.7 ± 1.9 mm). Testis length in the breeding season was significantly greater than in the non-breeding season (p < 0.01). **c** Comparison of the length of musk glands between the breeding and non-breeding seasons. The *left bar* represents the average length of the musk gland in April (38.3 ± 4.0 mm). The *right bar* represents the average length of the musk gland in November (15.3 ± 6.7 mm). The length of the musk gland in the breeding season was significantly greater than that in the non-breeding season (p < 0.01) (**p < 0.01). **d** Testis morphology in the breeding and non-breeding seasons. **e** Musk gland morphology in the breeding and non-breeding seasons. **f** Histological structure of the muskrat testis in the breeding season. **g** Histological structure of the muskrat testis in the non-breeding season. **h** Histological structure of the muskrat musk gland in the breeding season. **i** Histological structure of the muskrat musk gland in the non-breeding season. *GC* glandular cells, *EC* epithelial cells, *IC* interstitial cells, *LC* Leydig cells, *Spg* spermatogonium, *pSpc* primary spermatocyte, *rSpd* round spermatid, *eSpd* elongated spermatid

The weight of musk that the selected muskrats secreted during the breeding and non-breeding seasons was recorded and summed for the two seasons (Fig. 1a). The total weight produced in the breeding season (17.36 ± 1.67 g in March and April) was significantly higher than that in the non-breeding season (2.16 ± 0.40 g in October and November), (p < 0.01).

### The seasonal profile of musk gland and testis morphology

The lengths of musk glands during the two seasons were significantly different (Fig. 1c). It is obvious that the musk gland in the breeding (38.3 ± 4.0 mm) was much longer than in the non-breeding season (15.3 ± 6.7 mm, p < 0.01). Similar changes occurred in the testis (Fig. 1b), which was much larger in the breeding (20.6 ± 2.5 mm) than in the non-breeding season (10.7 ± 1.9 mm, p < 0.01). Morphological observations during the breeding and non-breeding seasons of musk glands and testis are compared in Fig. 1d, e. The seasonal differences were significant. Musk glands and testes were larger in the breeding season. The musk gland cells and epithelial cells become smaller in the non-breeding season. In the testis, the Leydig cells produce testosterone. The seminiferous tubules and Leydig cells were smaller in the non-breeding season. There were fewer developed spermatids in the non-breeding season.

### Immunohistochemical expression of AR in the musk gland

**Fig. 2:**
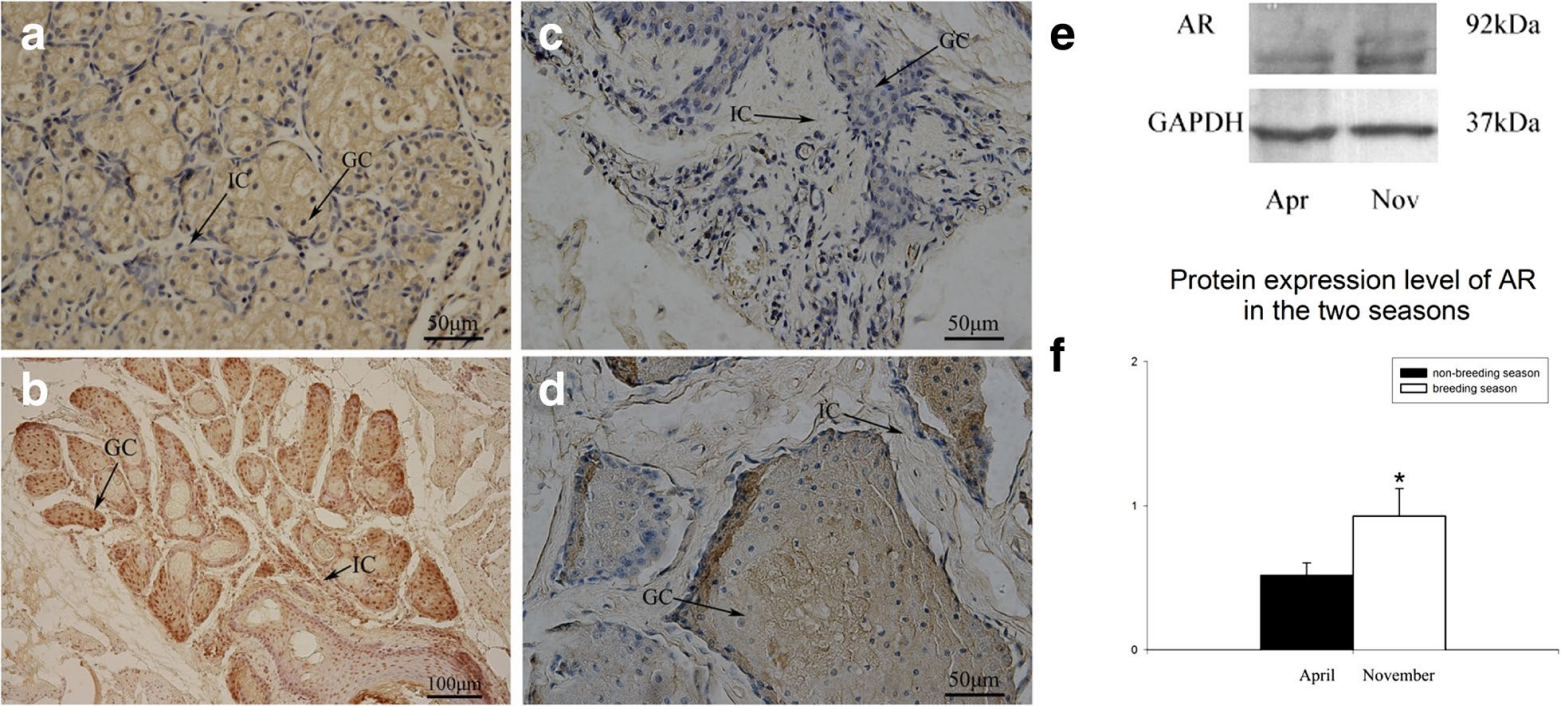
Immunohistochemistry and Western-blotting results of AR expression in musk glands. **a** Immunolocalization of androgen receptor (AR) in muskrat musk gland in the breeding season. **b** Immunolocalization of AR in the musk deer (*Moschus berezovskii*) musk gland in the breeding season. **c** Immunolocalization of AR in muskrat musk gland in the non-breeding season. **d** Immunolocalization of AR in the small Indian civet (*Viverricula indica*) musk gland in the breeding season. *GC* glandular cells, *IC* interstitial cells. **e** Positive bands of AR and GAPDH. **f** The comparison of AR protein expression between the two seasons. The expression level was the quotient of the values of AR and GAPDH. And the value of breeding season is higher than the value in non-breeding season (0.01 < p  <  0.05)

Androgen receptor was detected in the musk gland during the breeding and non-breeding seasons by immunohistochemistry (Fig. 2a, c), and localized mainly in the glandular cells. The positive section of breeding season was more significant. The distribution of AR in muskrat musk glands was similar to that in musk deer (Fig. 2b) and in the small Indian civet (Fig. 2d).

### Protein expression of AR in the musk gland

According to the Western blotting results (Fig. 2e), the positive bands of AR were shown at 92 kDa. We considered GAPDH as a reference which were shown at 37 kDa. The comparison of AR protein level in non-breeding and breeding season is significant (Fig. 2f).

### Immunohistochemical detection of StAR, P450scc and 3β-HSD in the testis

**Fig. 3:**
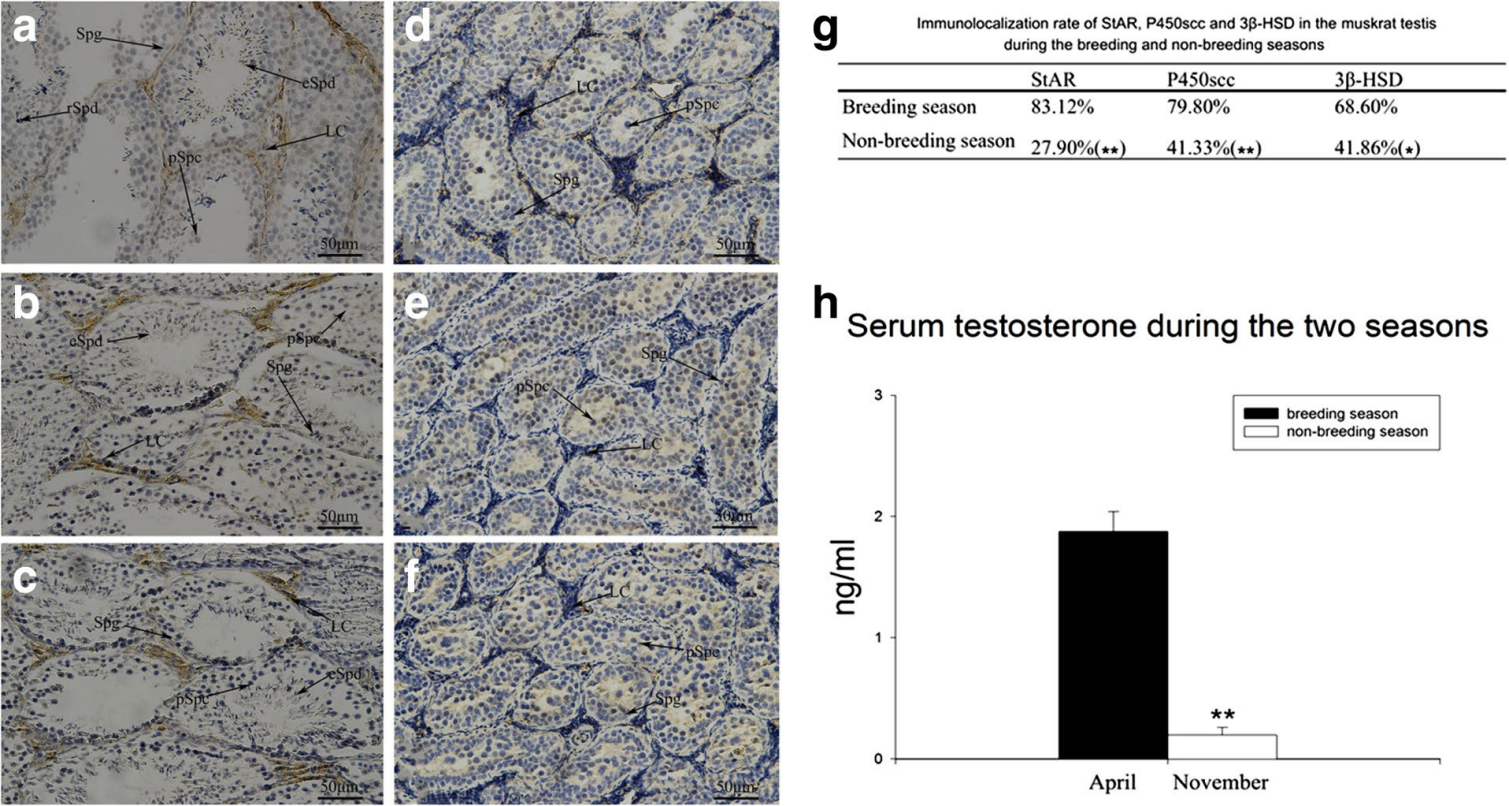
Immunohistochemistry results in testis. **a** Immunolocalization of StAR in the testis in the breeding seasons. **b** Immunolocalization of P450scc in the testis in the breeding seasons. **c** Immunolocalization of 3β-HSD in the testis in the breeding season. **d** Immunolocalization of StAR in the testis in the non-breeding seasons. **e** Immunolocalization of P450scc in the testis in the non-breeding seasons. **f** Immunolocalization of 3β-HSD in the testis in the non-breeding season. *LC* Leydig cells, *Spg* spermatogonium, *pSpc* primary spermatocyte, *rSpd* round spermatid, *eSpd* elongated spermatid. **g** The immunodetection rate of StAR, P450scc and 3β-HSD in the breeding season were all significantly higher than in the non-breeding season (0.01 < p < 0.05; **p < 0.01). **h** Testosterone in the serum samples was assayed by ELISA three times. The *left bar* represents the average testosterone concentration in the breeding season (1.874 ± 0.167 ng/ml). The *right bar* represents the average testosterone concentration in the non-breeding season (0.198 ± 0.067 ng/ml). The seasonal difference between the average serum testosterone concentrations was significant (p < 0.01). (**p < 0.01)

StAR, P450scc and 3β-HSD were detected in testis in the breeding and non-breeding seasons (Fig. 3a–f). Positive reaction for StAR, P450scc and 3β-HSD was observed mainly in the Leydig cells in both seasons. These three factors were significantly more highly expressed in the breeding than in the non-breeding season.

The immunodetection rate was calculated as the percentage of positive Leydig cells among the total number of Leydig cells. The immunodetection rates of StAR, P450scc and 3β-HSD were all reduced in the non-breeding season (Fig. 3g).

### Serum testosterone concentration

The concentrations of testosterone in the serum of muskrats in the breeding and non-breeding seasons were measured using an ELISA kit. There was a significant decrease in serum testosterone from the breeding (1.874 ± 0.167 ng/ml in April) to the non-breeding season (0.198 ± 0.0673 ng/ml in November, p < 0.01) (Fig. 3h).

### AR mRNA levels in the musk gland

**Fig. 4:**
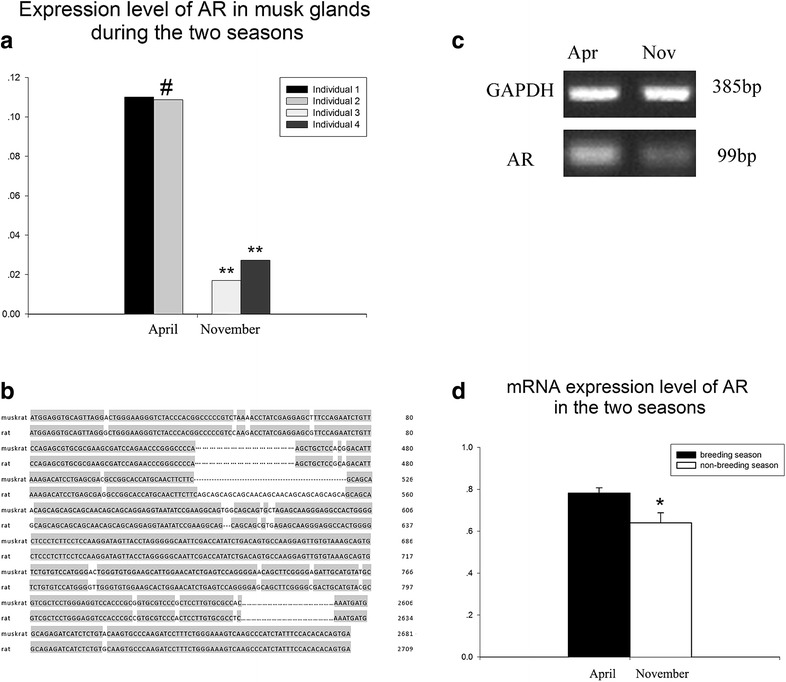
RNA-seq analysis and RT-PCR results of AR in musk glands. **a** The expression levels of two individuals in the same season were similar (p > 0.05), and the expression levels of two individuals in different seasons were significantly different (p < 0.01). (*p > 0.05; **p < 0.01) Individuals 1 and 2: breeding season. Individuals 3 and 4: non-breeding season. **b** Comparison of androgen receptor mRNA sequence of rat (*Rattus norvegicus*) and muskrat (*Ondatra zibethicus*). (*Dotted lines* represents for the sequence not mentioned on purpose; *straight line* represents for the missing sequence during the RNA-sequencing analysis; the complete CDS results were presented in Additional file 1: Figure S1). **c** Positive bands of AR and GAPDH. **d** The comparison of AR mRNA expression between the two seasons. And the value of breeding season is higher than the value in non-breeding season. (0.01 < p  <  0.05)

The expression levels of androgen receptor were assayed in 4 musk glands from 4 individuals from the breeding and non-breeding seasons (April and November) (Fig. 4a). The expression levels of AR in the breeding were significantly higher than in the non-breeding season (p < 0.01).

The expression of AR mRNA of breeding sample is stronger than that of non-breeding season sample (Fig. 4c). The comparison of AR mRNA level in non-breeding and breeding season is significant (Fig. 4d).

The antibodies used in this study were all polyclonal antibodies. Comparing the RNA-sequencing analysis results with the mRNA found in NCBI gene bank, we found that the CDS of androgen receptor mRNA from muskrat had 94.4% similarity with the CDS from rat (*Rattus norvegicus*). It is considered that the androgen receptors of rat and muskrat has high similarity (Fig. 4b).

## Discussion

The cyclic alternation between gonads growth and involution is a well-known phenomenon of seasonal breeders. The present study shows that total muskrat testis weights in the breeding were higher than in the non-breeding season (Fig. 1a). Muskrat testes were significantly larger in the breeding than in the non-breeding season (Fig. 1c). The musk glands were also significantly smaller in the non-breeding season (Fig. 1b). These seasonal changes suggest that testes and musk glands develop synchronously.

Changes in the seminiferous tubules from the breeding to the non-breeding season were also significant. It was difficult to find developed sperm cells in the seminiferous tubules of non-breeding muskrats (Fig. 1g). Similar observations have been made in other species. Findings in raccoon dogs [17], roe deer [18], horse [19], and ground squirrels [20] suggest that seasonal changes in testis size are correlated with changes in the numbers of germ cells in seasonal breeders. These results are consistent with the view that cyclical growth and involution of the testes may be universal in seasonal breeders [21]. In this study, we observed that there is also a cyclic alternation between growth and involution of the muskrat musk gland (Fig. 1h, i). This result is also in agreement with previously published work [2]. The morphological results suggest an important relationship between the muskrat musk gland and testis, simultaneously growing during the breeding season and involuting during the non-breeding season. Musk gland development and function might be regulated by the testis through testosterone produced in the Leydig cells.

Androgens have various important functions. The present study revealed that AR expression in the muskrat musk gland is higher in the breeding than in the non-breeding season (Fig. 2a, c). A previous study also detected AR in musk glands of muskrats [1, 2]. We also found AR distributed in the musk glands of small Indian civet and musk deer (Fig. 2b, d). According to the RNA-seq results AR expression in muskrat musk glands during the breeding season was significantly higher than during the non-breeding season (Fig. 4a). Additionally, AR mRNA and protein expression in the musk glands were both are higher in the breeding season (Figs. 2f, 4d). A similar trend also occurred in the serum testosterone concentration changes (Fig. 3h). These changes suggested that far more AR and androgens are needed in the breeding season to regulate musk gland development and musk production, which is in agreement with previous research [1, 2]. Androgens mainly function via the hypothalamus-pituitary-testis system to regulate testis development. In light of our current results, we propose that androgens may have the important function of regulating the seasonal co-development of testes and musk glands. The antibody used in this study was designed to test androgen receptor expression in rat, mouse, and human tissue.

Because of the seasonal involutional changes in the testis, the volume of seminiferous tubules and Leydig cells in the non-breeding season was predicted to be smaller than that in the breeding season (Fig. 1d–i). According to the results of the hormone analysis, these changes might be regulated by seasonal changes in serum testosterone concentration (Fig. 3h). Others have conducted similar research on the function of testosterone in testis development [22, 23]. It is clear that StAR, P450scc, and 3β-HSD in the Leydig cells regulate testosterone production [24]. The immunohistochemical observations of the three factors in the muskrat testis in the breeding and non-breeding seasons (Fig. 3a–f) provide new evidence that the Leydig cells’ regulatory function for producing androgens changes seasonally. The immunolocalization of StAR, P450scc, and 3β-HSD differed significantly in the breeding compared with the non-breeding season, particularly in the Leydig cells. The immunohistochemical results support the observed difference in serum testosterone concentrations between the two seasons. The levels of StAR, P450scc, and 3β-HSD as detected by immunohistochemistry were lower in the non-breeding season (Fig. 3g), indicating that the testes’ ability to produce testosterone was weaker in the non-breeding season, and this explains the seasonal difference in serum testosterone concentration.

## Conclusions

The mechanism of musk gland development and musk secretion need further study. Muskrat is an appropriate model to study on the mechanism. As similar species that secrete musk, civet and musk deer are protected by law. Muskrats can provide opportunities to conduct molecular study, while it can hardly be operated on protected animals.

**Fig. 5:**
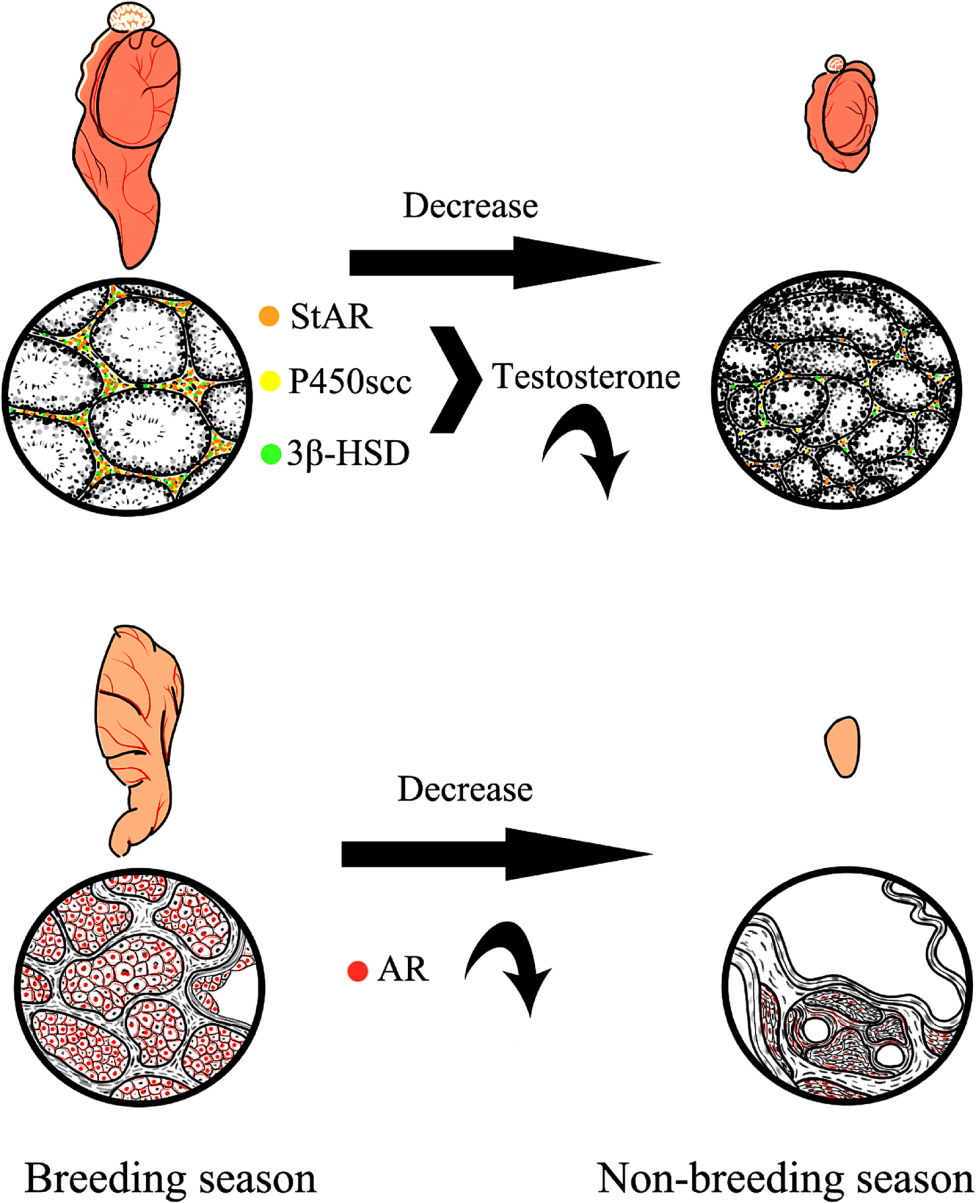
Sketch to show seasonal changes in musk gland and testis

The immunolocalizations of StAR, P450scc and 3β-HSD in testis express less in non-breeding season. While the immunolocations of AR in musk glands express weaker at the same time (Fig. 5). That is the reason testis’ ability to synthesize testosterone and the ability of musk gland to receive testosterone are weak in non-breeding season. In summary, this study presents new evidence that muskrat testes and musk glands exhibit synchronous seasonal growth and involution, AR have an important role in the connection of testis and musk glands (Fig. 5).

## Additional file

**Additional file 1: Figure S1.** CDS of androgen receptor in rat (*Rattus norvegicus*) and muskrat (*Ondatra zibethicus*).

## Abbreviations

AR: androgen receptor
StAR: steroidogenic acute regulatory protein
P450scc: cytochrome P450 cholesterol side-chain cleavage
3β-HSD: 3β-hydroxysteroid dehydrogenase
APES: 3-aminopropyl-triethoxysilane
ELISA: enzyme linked immunosorbent assay
RNA: ribonucleic acid
