# Supplementary material for: Musk gland seasonal development and musk secretion are regulated by the testis in muskrat (Ondatra zibethicus)
Source: Biol Res. 2017 Mar 4;50:10. doi: 10.1186/s40659-017-0116-9 (PMC5337303; doi:10.1186/s40659-017-0116-9)
Supplement: Supplementary file 1 — Additional file 1: Figure S1. CDS of androgen receptor in rat (Rattus norvegicus) and muskrat (Ondatra zibethicus). [file 40659_2017_116_MOESM1_ESM.docx]

muskrat ATGGAGGTGCAGTTAGGACTGGGAAGGGTCTACCCACGGCCCCCGTCTAAAACCTATCGAGGAGCTTTCCAGAATCTGTT 80

rat ATGGAGGTGCAGTTAGGGCTGGGAAGGGTCTACCCACGGCCCCCGTCCAAGACCTATCGAGGAGCGTTCCAGAATCTGTT 80

muskrat CCAGAGCGTGCGCGAAGCGATCCAGAACCCGGGCCCCAGGCACCCTGAGGCCGCAAGCACAGCACCTCCCGGTGCCTGTT 160

rat CCAGAGCGTGCGCGAAGCGATCCAGAACCCGGGCCCCAGGCACCCTGAGGCCGCTAGCATAGCACCTCCCGGTGCCTGTT 160

muskrat TGCAGCAGCGGCAAGAGACTAGCCCCCGGCGGCGGAGGCGGCAGCAGCACGGCGAGGATGGCTCTCCCCAAGCCCACATC 240

rat TACAGCAGCGGCAGGAGACTAGCCCCCGGCGGCGGCGGCGGCAGCAGCACCCTGAGGATGGCTCTCCTCAAGCCCACATC 240

muskrat AGAGGCCCCACAGGCTACCTGGCCCTGGAGGAAGAACAGCAACCTTCACAACAGCAGTCAGCCTCCGAGGGCCACCCTGA 320

rat AGAGGCACCACAGGCTACCTGGCCCTGGAGGAGGAACAGCAGCCTTCACAGCAGCAGTCAGCCTCCGAGGGCCACCCTGA 320

muskrat GAGCGGCTGCCTCCCAGAGCCTGGGACTGCTACAGCTCCTGGCAAGGGGCTGCCGCAGCAGCCACCAGCTCCTCCAGATG 400

rat GAGCGGCTGCCTCCCGGAGCCTGGAGCTGCCACGGCTCCTGGCAAGGGGCTGCCGCAGCAGCCACCAGCTCCTCCAGATC 400

muskrat AGGATGACTCAGCTGCCCCATCCACGTTGTCCCTGCTGGGCCCCACTTTCCCAGGCTTAAGCAGCTGCTCCACGGACATT 480

rat AGGATGACTCAGCTGCCCCATCCACGTTGTCCCTACTGGGCCCCACTTTCCCAGGCTTAAGCAGCTGCTCCGCAGACATT 480

muskrat AAAGACATCCTGAGCGACGCCGGCACCATGCAACTTCTTC----------------------------------GCAGCA 526

rat AAAGACATCCTGAGCGAGGCCGGCACCATGCAACTTCTTCAGCAGCAGCAGCAACAGCAACAGCAGCAGCAGCAGCAGCA 560

muskrat ACAGCAGCAGCAGCAACAGCAGCAGGAGGTAATATCCGAAGGCAGTGGCAGCAGTGCTAGAGCAAGGGAGGCCACTGGGG 606

rat GCAGCAGCAGCAGCAACAGCAGCAGGAGGTAATATCCGAAGGCAG---CAGCAGCGTGAGAGCAAGGGAGGCCACTGGGG 637

muskrat CTCCCTCTTCCTCCAAGGATAGTTACCTAGGGGGCAATTCGACCATATCTGACAGTGCCAAGGAGTTGTGTAAAGCAGTG 686

rat CTCCCTCTTCCTCCAAGGATAGTTACCTAGGGGGCAATTCGACCATATCTGACAGTGCCAAGGAGTTGTGTAAAGCAGTG 717

muskrat TCTGTGTCCATGGGACTGGGTGTGGAAGCATTGGAACATCTGAGTCCAGGGGAACAGCTTCGGGGAGATTGCATGTATGC 766

rat TCTGTGTCCATGGGGTTGGGTGTGGAAGCACTGGAACATCTGAGTCCAGGGGAGCAGCTTCGGGGCGACTGCATGTACGC 797

muskrat GTCGCTCCTGGGAGGTCCACCCGCGGTGCGTCCCGCTCCTTGTGCGCCACTGGCCGAATGCAAAGGTCTTCCCCTGGACG 846

rat GTCGCTCCTGGGAGGTCCACCCGCCGTGCGTCCCACTCCTTGTGCGCCTCTGGCCGAATGCAAAGGTCTTTCCCTGGACG 877

muskrat AGGGCCCGGGAAAAGGCACTGAAGAGACTGCTGAGTATTCCTCTTTCAAGGGAGGTTACGCCAAAGGGCTGGAAAGCGAG 926

rat AAGGCCCGGGCAAAGGCACTGAAGAGACTGCTGAGTATTCCTCTTTCAAGGGAGGTTACGCCAAAGGGTTGGAAGGTGAG 957

muskrat AGCCTGGTCTGCTCTGGCAGCGGTGAAGCAGGTAGTTCTGGGACACTTGAGATCCCGTCCACTCTGTCTCTGTATAAATC 1006

rat AGTCTGGGCTGCTCTGGCAGCAGTGAAGCAGGTAGCTCTGGGACACTTGAGATCCCGTCCTCACTGTCTCTGTATAAGTC 1037

muskrat CGGAGCAGTGGATGAAGCAGCAGCATACCAGAATCGCGACTACTACAACTTTCCACTGGCTCTGACCGGGCCGCCGCACC 1086

rat TGGAGCAGTAGACGAGGCAGCAGCATACCAGAATCGCGACTACTACAACTTTCCGCTCGCTCTGTCCGGGCCGCCGCACC 1117

muskrat CCCAGCCCCCTACCCATCCACACGCCCGCATCAAGCTGGAGAACCCTCTGGATTATGGCAGCGCCTGGGCTGCTGCGGCC 1166

rat CCCCGCCCCCTACCCATCCACACGCCCGCATCAAGCTGGAGAACCCGTCGGACTACGGCAGCGCCTGGGCTG---CGGCG 1194

muskrat GCGGCGCAATGCCGCTATGGAGACTTGGCTAGCCTGCACGGAGGGAGTGCAGTCGGACCCAGCACCGGATCGCCCCCAGC 1246

rat GCAGCGCAATGCCGCTATGGGGACTTGGCTAGCCTACATGGAGGGAGTGTAGCCGGACCCAGCACTGGATCGCCCCCAGC 1274

muskrat CACCTCCTCTTCTTCCTGGCACACTCTCTTCACAGCTGAAGAAGGTCAATTATATGGGCCAGGCGGAGGGGGCGACAGTA 1326

rat CACCGCCTCTTCTTCCTGGCATACTCTCTTCACAGCTGAAGAAGGCCAATTATATGGGCCAGGAGGCGGGGGCGGCAGCA 1354

muskrat GCAGCCCCAGCGAGGCAGGGCCTGTAGCTCCCTATGGCTACACTCGTCCCCCTCAGGGGCTGGCGAGTCAGGAGGGTGAC 1406

rat GTAGCCCAAGCGATGCTGGGCCTGTAGCCCCCTATGGCTACACTCGGCCCCCTCAGGGGCTGGCAAGCCAGGAGGGTGAC 1434

muskrat TTCTCTGCCTCCGAAGTGTGGTACCCCAGTGGAGTGGTGAACAGAGTACCCTATCCCAGTCCCAGTTACGTCAAAAGTGA 1486

rat TTCTCTGCCTCTGAAGTGTGGTATCCTGGTGGAGTTGTGAACAGAGTCCCCTATCCCAGTCCCAGTTGTGTTAAAAGTGA 1514

muskrat AATGGGACCTTGGACGGAGAACTACTCCGGACCTTATGGAGACATGCGTTTGGACAGTGCCAGGGACCACGTTTTACCCA 1566

rat AATGGGACCTTGGATGGAGAACTACTCCGGACCTTATGGGGACATGCGTTTGGACAGTACCAGGGACCACGTTTTACCCA 1594

muskrat TCGACTATTACTTTCCACCCCAGAAGACCTGCCTGATCTGCGGCGACGAAGCTTCTGGCTGTCACTATGGAGCCCTCACT 1646

rat TCGACTATTACTTCCCACCCCAGAAGACCTGCCTGATCTGTGGAGATGAAGCTTCTGGTTGTCACTACGGAGCTCTCACT 1674

muskrat TGTGGCAGCTGCAAGGTCTTCTTCAAAAGAGCCGCTGAAGGGAAGCAGAAATATCTGTGTGCCAGCAGAAATGATTGTAC 1726

rat TGTGGCAGCTGCAAGGTCTTCTTCAAAAGAGCTGCGGAAGGGAAACAGAAGTATCTATGTGCCAGCAGAAATGATTGCAC 1754

muskrat CATTGATAAATTTCGGAGGAAGAATTGTCCATCTTGTCGACTCCGGAAATGTTATGAAGCAGGGATGACTCTGGGAGCTC 1806

rat CATTGATAAATTTCGGAGGAAAAATTGTCCATCGTGTCGTCTCCGGAAATGTTATGAAGCAGGGATGACTCTGGGAGCTC 1834

muskrat GTAAGCTGAAAAAACTTGGAAATCTAAAACTACAGGAGGAAGGAGAAAACTCCAATGCTGCTAGCCCCACTGAGGACCCA 1886

rat GTAAGCTGAAGAAACTTGGAAATCTCAAACTACAGGAAGAAGGAGAAAACTCCAGTGCTGGTAGCCCCACTGAGGACCCA 1914

muskrat TCCCAGAAGATGACAGTATCACACATTGAAGGCTATGAATGTCAACCTATCTTTCTTAATGTCCTGGAAGCTATTGAACC 1966

rat TCCCAGAAGATGACTGTATCACACATTGAAGGCTATGAATGTCAACCTATCTTTCTTAATGTCCTGGAAGCCATTGAGCC 1994

muskrat AGGAGTGGTGTGTGCTGGACATGACAACAACCAGCCGGATTCCTTTGCTGCCTTGTTATCTAGCCTCAATGAGTTGGGCG 2046

rat AGGAGTGGTGTGTGCCGGACATGACAACAACCAGCCTGATTCCTTTGCTGCCTTGTTATCTAGTCTCAACGAGCTTGGCG 2074

muskrat AGAGACAGCTTGTACATGTGGTCAAGTGGGCCAAGGCCTTGCCTGGATTCCGCAACTTGCATGTAGATGACCAGATGGCA 2126

rat AGAGACAGCTTGTACATGTGGTCAAGTGGGCCAAGGCCTTGCCTGGCTTCCGCAACTTGCATGTGGATGACCAGATGGCA 2154

muskrat GTCATTCAGTATTCCTGGATGGGACTCATGGTATTTGCCATGGGTTGGCGGTCCTTCACTAACGTCAACTCCAGGATGCT 2206

rat GTCATTCAGTATTCCTGGATGGGACTGATGGTATTTGCCATGGGTTGGCGGTCCTTCACTAATGTCAACTCTAGGATGCT 2234

muskrat CTACTTTGCACCTGACCTGGTTTTCAATGAGTACCGCATGCACAAGTCTCGGATGTACAGCCAGTGTGTGCGGATGAGGC 2286

rat CTACTTTGCACCTGACCTGGTTTTCAATGAGTATCGCATGCACAAGTCTCGAATGTACAGCCAGTGCGTGAGGATGAGGC 2314

muskrat AACTGTCTCAAGAATTCGGATGGCTCCAGATAACCCCCCAGGAATTCCTGTGTATGAAAGCCCTGCTACTCTTCAGCATT 2366

rat ACCTTTCTCAAGAGTTTGGATGGCTCCAGATAACCCCCCAGGAATTCCTGTGCATGAAAGCACTGCTACTCTTCAGCATT 2394

muskrat ATTCCAGTGGATGGGCTGAAAAATCAAAAATTCTTTGATGAACTTCGAATGAACTACATCAAGGAACTCGATCGTATCAT 2446

rat ATTCCAGTGGATGGGCTGAAAAATCAAAAATTCTTTGATGAACTTCGAATGAACTACATCAAGGAACTTGATCGCATCAT 2474

muskrat TGCATGCAAAAGAAAAAATCCCACATCCTGCTCAAGGCGCTTCTACCAGCTCACCAAGCTCCTGGATTCTGTGCAGCCTA 2526

rat TGCATGCAAAAGAAAAAATCCCACATCCTGCTCAAGGCGCTTCTACCAGCTCACCAAGCTCCTGGATTCTGTGCAGCCTA 2554

muskrat TTGCAAGAGAGCTGCATCAGTTCACTTTTGACCTGCTAATCAAGTCCCATATGGTGAGCGTGGACTTTCCTGAAATGATG 2606

rat TTGCAAGAGAGCTGCATCAATTCACTTTTGACCTGCTAATCAAGTCCCATATGGTGAGCGTGGACTTTCCTGAAATGATG 2634

muskrat GCAGAGATCATCTCTGTACAAGTGCCCAAGATCCTTTCTGGGAAAGTCAAGCCCATCTATTTCCACACACAGTGA 2681

rat GCAGAGATCATCTCTGTGCAAGTGCCCAAGATCCTTTCTGGGAAAGTCAAGCCCATCTATTTCCACACACAGTGA 2709
